# On the stress potential of videoconferencing: definition and root causes of Zoom fatigue

**DOI:** 10.1007/s12525-021-00501-3

**Published:** 2021-12-06

**Authors:** René Riedl

**Affiliations:** 1 School of Business and Management, Digital Business, University of Applied Sciences Upper Austria, Wehrgrabengasse 1-3, 4400 Steyr, Austria; 2 Institute of Business Informatics – Information Engineering, Johannes Kepler University Linz, Altenberger Strasse 69, Linz, 4040 Austria

**Keywords:** Zoom fatigue, Videoconference stress, Videoconference fatigue, Technostress, Media naturalness theory, NeuroIS, Home office, M1, I12, O3

## Abstract

As a consequence of lockdowns due to the coronavirus disease (COVID-19) and the resulting restricted social mobility, several billion people worldwide have recently had to replace physical face-to-face communication with computer-mediated interaction. Notably, the adoption rates of videoconferencing increased significantly in 2020, predominantly because videoconferencing resembles face-to-face interaction. Tools such as Zoom, Microsoft Teams, and Cisco Webex are used by hundreds of millions of people today. Videoconferencing may bring benefits (e.g., saving of travel costs, preservation of environment). However, prolonged and inappropriate use of videoconferencing may also have an enormous stress potential. A new phenomenon and term emerged, *Zoom fatigue*, a synonym for videoconference fatigue. This paper develops a definition for Zoom fatigue and presents a conceptual framework that explores the major root causes of videoconferencing fatigue and stress. The development of the framework draws upon media naturalness theory and its underlying theorizing is based on research published across various scientific fields, including the disciplines of both behavioral science and neuroscience. Based on this theoretical foundation, hypotheses are outlined. Moreover, implications for research and practice are discussed.

## Introduction

*Julia, a 32-year old social media consultant in a marketing agency, has to communicate much in personal meetings and via telephone, both with colleagues and clients. However, as a consequence of the COVID-19 crisis and restricted social mobility, she has been working most of the time from home since March 2020. With the beginning of the crisis, her employer quickly implemented Zoom videoconferencing software, and much of the original face-to-face and telephone communication has since been carried out *via* Zoom. Despite the fact that Julia acknowledges that she and her employer benefit from Zoom usage, she increasingly has mixed feelings about videoconferencing. On more and more days, most of which are dominated by videoconferences, she feels exhausted, fatigued, and stressed—“it’s like a drain of cognitive resources,” she says, when providing an introspective account of her current sentiment. Many people share her experiences and, therefore, have begun to consider videoconferencing as a new source of stress.*

As illustrated by this vignette, videoconferencing has been quickly adopted by many people and organizations as a response to the restricted social mobility that resulted from COVID-19-induced lockdowns. Despite the fact that videoconferencing has been available to the general public for about two decades (Skype, for example, was launched in 2003), adoption rates of tools increased dramatically starting in spring 2020 (Gartner, 2020). Systems such as Zoom have been implemented to maintain communication in various areas, including business, education, health care, science, various private domains, and even in legislation and judiciary (e.g., Fouda, 2020; Puddister & Small, 2020; Toney et al., 2021). As a result of the availability of videoconferencing, people and organizations have been able to maintain communication, thereby helping economies and societies to continue functioning. Use of videoconferencing also saves travel costs (e.g., Denstadli, 2004; Denstadli et al., 2012) and contributes to the preservation of the environment (e.g., Aguilera, 2008).

The problem, however, is that the radical adoption and extensive use of videoconferencing tools also has a dark side, referred to as Zoom fatigue. This stress-related depletion of physiological and cognitive resources is a consequence of a prolonged and inappropriate use of videoconferencing tools. Note that Zoom fatigue is used as a synonym for videoconference fatigue, and hence also applies to the exhaustion that may result from the use of other similar tools.^1^

With the enormous increase of videoconferencing adoption rates in Spring 2020, newspaper and magazine reports emerged that used the term *Zoom fatigue* (e.g., Fosslien & Duffy, 2020; Morris, 2020; Sklar, 2020). Also, scientists in various disciplines including psychology (Wiederhold, 2020), Information Systems (IS) (Toney et al., 2021), human–computer interaction (Bailenson, 2021), psychophysiology (Peper et al., 2021), and health science (Brown Epstein, 2020) began to describe this new phenomenon. Recent survey evidence substantiates the significance of the problem (e.g., Asgari et al., 2021; Fauville et al., 2021b; Rump & Brandt, 2020a, 2020b). Moreover, recently the journal *Australasian Psychiatry* published a short paper entitled “Chronic Zoom Syndrome” in which the authors write about “a new diagnosis of paramount significance […] which may be included in international diagnostic classifications [and this] proposed diagnosis is based on clinical observations of an insidious and debilitating video-meeting-mediated disorder” (Anderson & Looi, 2020, p. 669).

Against the background of these recent developments, both in practice and science, there is an urgent need to explore the new phenomenon called Zoom fatigue in more detail, in particular its root causes. This urgency is substantiated by the fact that both the e-collaboration and technostress literature, two major IS research streams in which videoconference fatigue and stress as well as possible root causes should actually be a relevant phenomenon, have been completely silent about this issue so far.

The article is structured as follows. In Sect. 2, we outline the research gap in more detail. In Sect. 3, we develop a Zoom fatigue definition. In Sect. 4, we outline the study context and describe media naturalness theory (Kock, 2004, 2005, 2009a) as the theoretical lens through which six root causes of Zoom fatigue are derived. Section 5 discusses each of the six root causes in detail. Based on the theoretical understanding of the phenomenon that is summarized in a conceptual framework with corresponding hypotheses, Sect. 6 describes implications for research and practice, as well as limitations. In Sect. 7, a concluding statement is provided.

Methodologically, this paper comprises two approaches. First, the development of a Zoom fatigue definition in Sect. 3 draws upon a systematic literature review. Second, the development of the conceptual framework in Sect. 5 uses media naturalness theory as overarching model and draws upon published theoretical and empirical works from both the behavioral and neuroscience disciplines. Thus, a NeuroIS approach (Dimoka et al., 2012; Riedl & Léger, 2016) based on secondary sources along with deductive reasoning is used to substantiate the rationale and arguments provided. The usefulness of neuroscience evidence for IS theorizing without directly using neuroscience tools is a widely accepted approach in IS research (Dimoka et al. 2012; Riedl et al., 2017). As substantiation for a NeuroIS approach in the present videoconference stress context, we cite vom Brocke et al. (2020) who have made a call for NeuroIS research recently and indicate that “topics on the individual level, such as stress, are considered particularly suitable for NeuroIS” (p. 10); they further outline that “usage of digital communication devices” and “digital communication’s inability to send the full range of non-verbal signals” (p. 24) are topics of societal relevance that should also be examined through a neuroscience lens.

## Outline of the research gap and contribution

Electronic collaboration (e-collaboration) is a term that refers to all computer-based modes, across distributed contexts, that support interaction, communication, and coordination among people (Riemer, 2009). E-collaboration research may pertain to different levels of analysis, including individual, team (group), organization, and society (Gallivan & Benbunan-Fich, 2005). Videoconferencing is an important topic on the individual and group levels. Analysis of the literature indicates that this topic has become an important subject of study over the years; application domains of videoconferencing which have been studied scientifically are, among others, business (e.g., Graetz et al., 1998; Hambley et al., 2007; Maynard & Gilson, 2014), education (e.g., Giesbers et al., 2013; Padilla-Meléndez et al., 2008), and health care (e.g., Barton et al., 2011; Mair & Whitten, 2000).

However, inspection of the e-collaboration literature, including the literature on videoconferencing and virtual teams, reveals that except one single study (Wegge et al., 2007) no peer-reviewed academic paper exists with a focus on the stress potential of videoconferencing. In this study, psychologists simulated video-based call center work in an experiment and found that time pressure of call center agents causes strain, and that this relationship is moderated by customer friendliness. Thus, direct examination of the fatigue and stress potential of videoconferencing itself was not the focus of this study. Further substantiation of the research gap comes from a recent review of the virtual teams literature that already considers COVID-19 developments (Kilcullen et al., 2021). Surprisingly, this review does not even mention the terms “stress”, “fatigue”, “strain”, and “exhaustion”. Against this background, we conclude that the e-collaboration literature, including the literature on videoconferencing and virtual teams, has not yet examined the stress potential of videoconferencing.

Additionally, we also analyzed the technostress literature. However, it has not studied videoconference stress either (Benzari et al., 2020). In fact, the most recent review article on technostress (Grummeck-Braamt et al., 2021; *N* = 252 papers) and five existing reviews referenced in this article (Fischer & Riedl, 2015; Fischer & Riedl, 2017; La Torre et al., 2019; Riedl, 2013; Tarafdar et al., 2019) have been *completely silent* about the phenomenon. No single word related to videoconference stress and Zoom fatigue can be found in these six reviews *and* their underlying literature basis (in total several hundreds of articles). Because technostress refers to stress that results from the use of information and communication technologies (ICTs) (Ayyagari et al., 2011; Ragu-Nathan et al., 2008), of which videoconferencing is an important instance, this finding is remarkable.

A possible reason for this research gap is that adoption rates were not high before the COVID-19 pandemic and hence videoconferencing, if compared to other technologies, did not play a significant role. However, this situation has changed sharply within a short period of time, as signified by recent findings of IS studies which were conducted in the context of COVID-19 induced lockdowns. For example, Hacker et al. (2020) report that “the heavy reliance on web-conferencing as the main medium for conducting one’s life led to physical and mental exhaustion” (p. 578). In another recent study, Waizenegger et al. (2020) indicate that “participants suffered from ‘virtual meetings-fatigue’ as virtual meetings are far more attention-taxing than face-to-face meetings” (p. 435). Moreover, they indicate that Zoom fatigue is “a big issue [one that is] a lot bigger than face-to-face fatigue in meetings” (p. 436).

Considering that it is a well-established fact in the literature that various forms of technostress may have severe effects on physiological arousal, health, mental well-being, emotional exhaustion, depression, burnout, performance, productivity, job satisfaction, and organizational commitment (e.g., Benlian, 2020; Riedl, 2013; Tarafdar et al., 2019), conceptualizing Zoom fatigue and examining its root causes is critical. This does not only stimulate further theoretical and empirical research. Rather, based on a better understanding of the phenomenon it is also possible to suggest and design effective coping strategies and countermeasures, which, in turn, help to mitigate or avoid negative effects. This better understanding is even more important when considering that increasingly more evidence indicates that home office and the resulting high adoption rates of videoconferencing will also play a major role in the post-COVID era (Despujol et al., 2020).

## What is Zoom fatigue?

### Methodology of the literature review

In order to identify definitions of Zoom fatigue, a systematic literature review was conducted.^2^ The search process was based on existing recommendations, in particular vom Brocke et al. (2009). The main keyword used was “Zoom fatigue”. No publication year restriction was used for all searches. We only considered sources in English language. As outlined in detail below, the present review covers literature published before and on May 1, 2021. Note that additional keywords were used during the search process, namely: “videoconferenc* stress”, “videoconferenc* fatigue”, and “videoconferenc* exhaustion”. However, no further relevant papers could be identified based on these keywords.^3^ Therefore, the following findings refer to the keyword “Zoom fatigue”.

Step_1_: The search was started via Web of Science and Scopus starting on 12/02/2020 and a last query was made on 05/02/2021. This method resulted in the following number of hits: Web of Science (specification was topic) = 6 papers; Scopus (based on the default mode that covers title, abstract, and keywords) = 18 papers. Thus, the total number of articles was 24. After removing 6 duplicates (Abdelrahman, 2021; Collins, 2020; Ebner & Greenberg, 2020; Petriglieri, 2020; Chawla, 2021; Wiederhold, 2020) and 1 non-English paper (Dolezel 2020), we ended up with 17 unique articles (Step_1_ = 17).

Step_2_: In order to identify further relevant papers beyond articles published in Web of Science and Scopus, a search based on Harzing’s “Publish or Perish” software (version 7.31 Windows GUI edition) resulted in 42 hits (specification title word, database specification: Google Scholar, last query: 05/02/2021). Because 4 papers were duplicates (3 × Sander & Bauman, 2020, 1 × Wiederhold, 2020), 3 were non-English articles (Karabasz, 2020; Kuntardi, 2021; Pustikasar & Fitriyanti, 2021), and 1 article was a withdrawn paper (Bullock et al., 2021), we ended up with 34 unique articles (Step_2_ = 34).

The following 7 articles were identified in Step_1_ and Step_2_: Chawla (2021), Cranford (2020), Hall (2020), Nadler (2020), Petriglieri (2020), Toney et al. (2021), and Wiederhold (2020). Thus, the number of unique articles at this stage was 44 [17 (Step_1_) + 34 (Step_2_) – 7 (Step_1_ ∩ Step_2_)].

**Fig. 1:**
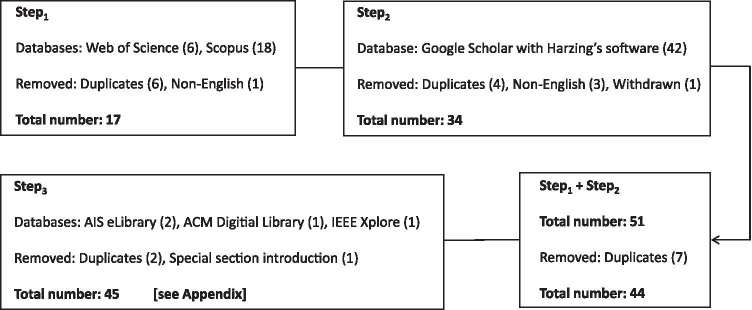
Overview of the literature search process

Step_3_: In order to identify further relevant papers beyond articles identified in Step_1_ and Step_2_, a search based on AIS eLibrary, ACM Digital Library, and IEEE Xplore was conducted (last query: 05/02/2021). The goal of this step was to identify additional papers, in particular articles with a focus on IS and computer science. The search via these three databases yielded the following results (based on search in title and abstract): AIS (2 hits: Toney et al., 2021 and a special section introduction that was referencing to Toney et al.), ACM (1 hit: Palti & Rosenberg-Kima, 2021), and IEEE (1 hit: Pesce, 2020). Because Toney et al. (2021) and Pesce (2020) had already been identified in a prior step and because the special section introduction was also removed, the number of unique relevant articles at this stage was 45. Backward and forward search did not yield further relevant papers. This is plausible because the phenomenon existed approximately only one year at the time the review was conducted and the searches itself already covered six large databases. Figure 1 graphically summarizes the literature search process.

### Zoom fatigue definition

The 45 articles were read to identify definitions of the term “Zoom fatigue”. In 12 publications a definition was identified (Abdelrahman, 2021; Anderson & Looi, 2020; Dixon-Saxon, 2020; Ebner & Greenberg, 2020; Fauville et al., 2021a; Hines & Sun, 2020; Lee, 2020; Miller, 2020; Nadler, 2020; Rump & Brandt, 2020a; Schroeder, 2020; Wiederhold, 2020).^4^ Thus, we ended up with a total of 12 unique definitions (see Table 1).

**Table 1:** Definitions of Zoom fatigue

Reference (alphabetical order)	Definition
1. Abdelrahman (2021, p. 11)	“[a] short hand for symptoms associated with all video conferencing technology […] an ‘exhausting ordeal’ that leaves the individual feeling mentally and physically wiped out. Its symptoms reportedly include headaches and migraines, blurred and double vision, eye irritation and pain, lack of focus and general exhaustion”
2. Anderson and Looi (2020, p. 669)	“an insidious and debilitating video-meeting-mediated disorder”
3. Dixon-Saxon (2020, p. 13)	“physical and mental exhaustion that results from information processing while on videoconferencing”
4. Ebner and Greenberg (2020, p. 537)	“physical and mental exhaustion that results from spending extended time videoconferencing”
5. Fauville et al., (2021a, p. 2)	“a feeling of exhaustion from participating in video conference calls”
6. Hines and Sun (2020, p. 1)	“the mental exhaustion associated with online video conferencing”
7. Lee (2020, p. 1)	“the tiredness, worry, or burnout associated with overusing virtual platforms of communication”
8. Miller (2020, p. 1)	“the feeling of tiredness, anxiousness or worry with yet another video call”
9. Nadler (2020, p. 2)	“a pan-descriptor for the symptoms people experience after prolonged technology use—typically CMC [computer-mediated communication] platforms with AVT [audio-visual technology]”
10. Rump and Brandt (2020a, p. 2)	“the fatigue that occurs after numerous virtual meetings during the day and over the week”
11. Schroeder (2020, p. 1)	“an array of physical and psychological factors that combine to make our synchronous online communications less effective and wrought with discomfort”
12. Wiederhold (2020, p. 437)	“tiredness, anxiety, or worry resulting from overusing virtual videoconferencing platforms”

The Appendix lists all 45 articles and characterizes them based on various criteria. Among others, this characterization reveals that the term “Zoom fatigue” emerged in the beginning of April 2020.

Based on the definitions in Table 1, we can identify the following immanent characteristics of Zoom fatigue:

- it refers to the *negative aspects of videoconferencing in general* (all definitions except #7, because “virtual platforms of communication” are not necessarily audio-visual technology, as in the example of pure instant messaging),
- it is associated with *long and repeated use of videoconferencing tools* (definitions #4, #7, #8, #9, #10, #12).
- it concerns both *physical and mental exhaustion* (definitions #1, #3, #4, #11), and
- it is linked to *similar phenomena such as tiredness, worry, anxiety, burnout, discomfort, and stress* (definitions #7, #8, #11, #12) and other bodily symptoms such as headaches (definition #1).^5^

Using an integrative consideration of these characteristics, we can develop the following definition:

*Zoom fatigue (synonym: videoconference fatigue) is defined as somatic and cognitive exhaustion that is caused by the intensive and/or inappropriate use of videoconferencing tools, frequently accompanied by related symptoms such as tiredness, worry, anxiety, burnout, discomfort, and stress, as well as other bodily symptoms such as headaches.*

## Study context and media naturalness theory as theoretical lens

### Study context

**Fig. 2:**
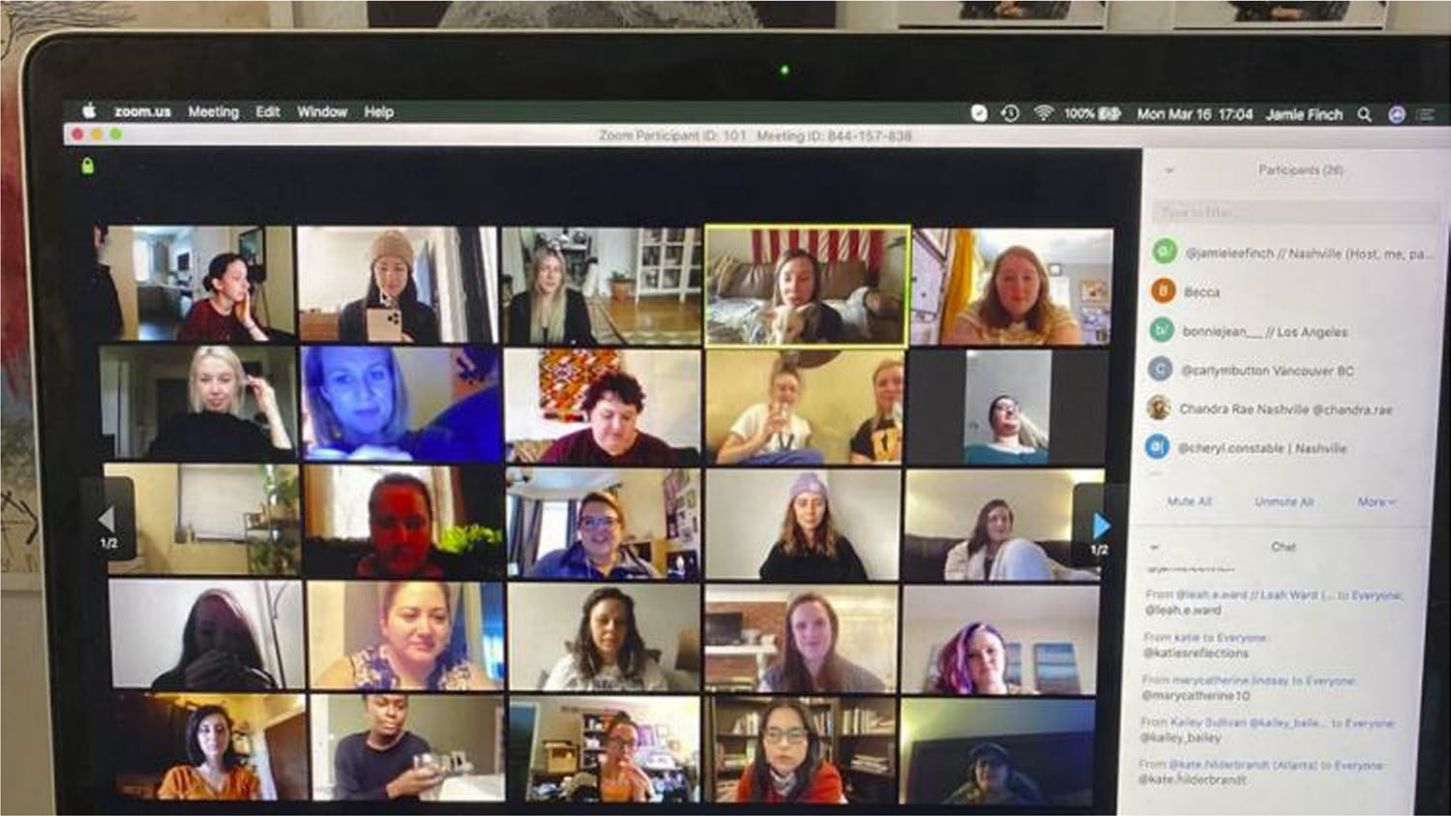
Typical use scenario of a videoconferencing tool (Picture: dpa, cited after Sueddeutsche (https://www.sueddeutsche.de/digital/zoom-fatigue-videokonferenz-ermuedung-corona-1.4888670))

Today many videoconferencing contexts comprise situations in which several people interact, as illustrated in Fig. 2. Examples are online seminars at universities or project team meetings in companies. Such a situation can be characterized by several attributes. Despite the fact that people can see and hear each other, they are not in the same location and the videoconference participants are not fully visible; typically only the face and sometimes part of the torso are visible. Due to low-quality cameras, different camera positions, and gazes that are often not directed toward the camera, perception of facial expressions is difficult, in many cases impossible. Moreover, the user’s own face is usually also visible on the screen. Finally, an inherent property of videoconferencing is that a number of faces are shown on the screen.

### Media naturalness theory

Biological anthropologists indicate that face-to-face (F2F) interaction has been the primary communication mode for more than 99% of human history (Boaz & Almquist, 2001; Cartwright, 2000). In light of Darwin’s (1859) theory of evolution, it is logical to conclude that the human trait of processing information in F2F situations must be part of the genetic makeup of humans. In fact, empirical evidence from various experimental paradigms (e.g., Goren et al., 1975; Willis & Todorov, 2006), along with theoretical arguments in the scientific literature, substantiate this notion. Kock (2005), for example, argues that “our brain has likely been to a large extent hardwired [i.e., genetically predetermined] for co-located and synchronous communication” (p. 120).

This innate human preference toward co-located F2F interaction suggests that people are *not* predisposed to communicate via electronic channels, even if a communication mode resembles F2F, as is the case with videoconferencing. This idea of an innate human preference toward co-located F2F interaction in the context of electronic media use has been formally described as media naturalness hypothesis (Kock, 2005), later renamed as media naturalness theory (Kock, 2009a). This theoretical framework defines the mismatch between the characteristics of F2F interaction and the characteristics of other modes of electronic communication (e.g., videoconferencing, e‑mail) as independent variables. Communication ambiguity and cognitive effort are major dependent variables. Considering these properties of media naturalness theory, it is evident that this theory constitutes a highly suitable conceptual lens through which major root causes of Zoom fatigue can be identified.

The degree of naturalness of a communication medium is assessed via the degree to which it incorporates the characteristics of F2F interaction (Kock, 2004, 2009a), namely: (1) the communicating individuals share the same context, and they are able to see and hear each other, (2) they can quickly exchange communicative stimuli (i.e., in real time), (3) the situation provides the ability to both convey and observe facial expressions, (4) to convey and observe body language, and (5) to convey and listen to speech. The theory predicts that a decrease in the degree of naturalness leads to an increase in communication ambiguity and cognitive effort. Moreover, as reviewed in Kock (2004) and Kock (2009a), a decrease in the degree of naturalness frequently has a negative effect on satisfaction, performance, and productivity for a number of collaborative tasks, despite the fact that humans also have the ability to compensate for lower degrees of naturalness in computer-mediated communication, which is referred to as compensatory adaptation (Carlson & Zmud, 1999; Kock, 2004).

Table 2 shows the results of an assessment of videoconferencing based on the five F2F characteristics and also identifies, based on this assessment, a main root cause of Zoom fatigue (*asynchronicity of communication*, *lack of body language*, *lack of eye contact*). These root causes, along with three further root causes that we derive in the following, are used as constructs in the conceptual framework (see Sect. 5).

**Table 2:** Assessment of videoconferencing based on the five F2F characteristics and root causes of Zoom fatigue

Characteristics of Face-to-Face	Fulfilled?	Comment	Major root cause of Zoom fatigue
(1) Communicating individuals share the same context, and they are able to see and hear each other	Partly	… because they do not share the same context (every participant is located in his or her individual context, e.g., home office)	Lack of eye contact
(2) Communicating individuals can quickly exchange stimuli (i.e., in real time)	Yes	… but full synchrony does not exist, because even perfectly working networks cannot function without a delay; humans are able to perceive delays of 200 ms in human–computer interaction (Kohrs et al., 2016; Miller, 1968)	Asynchronicity of communication
(3) The situation provides the ability to both convey and observe facial expressions	Partly	… depending on camera quality, size of the face on the screen, gaze direction, and camera angle, the situation is not the same as in co-located F2F interaction	Lack of eye contact (note that eye contact, if compared to information on muscles movements in the face, is more critical for successful coordination in human social interaction; Richardson et al., 2007; Saito et al., 2010)
(4) The situation provides the ability to convey and observe body language	No	… because videoconferencing, except in very unusual circumstances, does not include full body visualization	Lack of body language
(5) The situation provides the ability to convey and listen to speech	Yes	… but transmission delays may occur, caused by poor network quality and possible data transfer limitations	Asynchronicity of communication

The analysis in Table 2 shows that videoconferencing does not have the same characteristics as co-located F2F communication (despite the fact that at first glance might falsely indicate that it does). Characteristic #4 applies only to co-located F2F interaction. Moreover, while characteristics #2 and #5 are fulfilled, characteristics #1 and #3 are only partly fulfilled. It is important to keep in mind, as well, that the assessment in Table 2 assumes ideal technical conditions. However, evidence shows that technical issues such as latencies are a common problem in videoconferencing (Rump & Brandt, 2020a).

Media naturalness theory also predicts that an enrichment of human interaction through software features may lead to information overload (Kock, 2004). Therefore, an enrichment of electronic interaction through software features that create unnatural perceptions (i.e., for which the human brain has not been shaped by evolution) may lead to negative consequences, particularly information overload and increased cognitive effort (Kock, 2004, 2009a, 2009b). Videoconference systems such as Zoom incorporate software features which have the potential to create unnatural perceptions: *a window in which a user can see her- or himself (mirror effect)*, *a grid-view of other meeting participants based on which users get the feeling of unnatural interaction with multiple faces*, and *features that enforce people to multitask such as parallel processing of information provided simultaneously via the videostream and the chat function* (note that videoconference system users often engage in further parallel activities based on other programs). Hence, these features constitute potential root causes of Zoom fatigue.

**Fig. 3:**
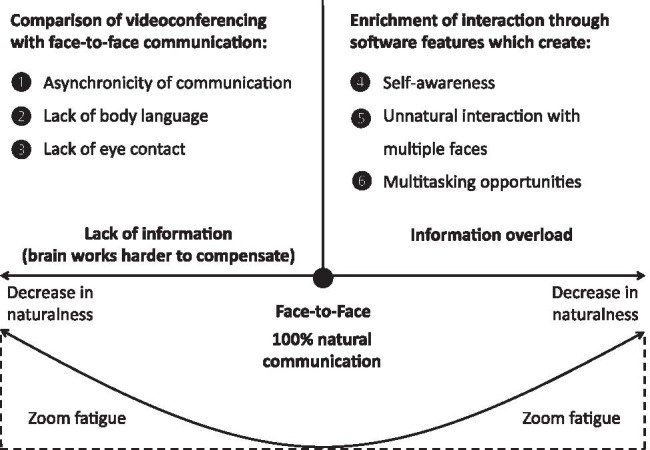
Decreases in naturalness of videoconferencing and Zoom fatigue

Figure 3 graphically summarizes the rationale why a decrease in a communication medium’s naturalness, here videoconferencing, leads to increased cognitive effort, and hence to Zoom fatigue. As shown on the left side, videoconferencing implies asynchronicity of communication, lack of body language, and lack of eye contact. Thus, if compared to F2F interaction, lack of information exists. Because the brain has a natural tendency to compensate for missing information by increased computations, lack of information that a person’s brain perceives in the course of social interaction—ironically—may cause increased cognitive effort (e.g., Frith & Frith, 2006; Lieberman, 2007; Satpute & Lieberman, 2006; Singer, 2009). Moreover, as shown on the right side, enrichment of videoconference interaction through software features may also lead to increased cognitive effort, a prediction that also follows from media naturalness theory (Kock, 2004, 2009a, 2009b).

Based on this description of media naturalness theory as a theoretical lens to study the root causes of Zoom fatigue, in the next section we develop the theoretical framework.

## Root causes of Zoom fatigue

**Fig. 4:**
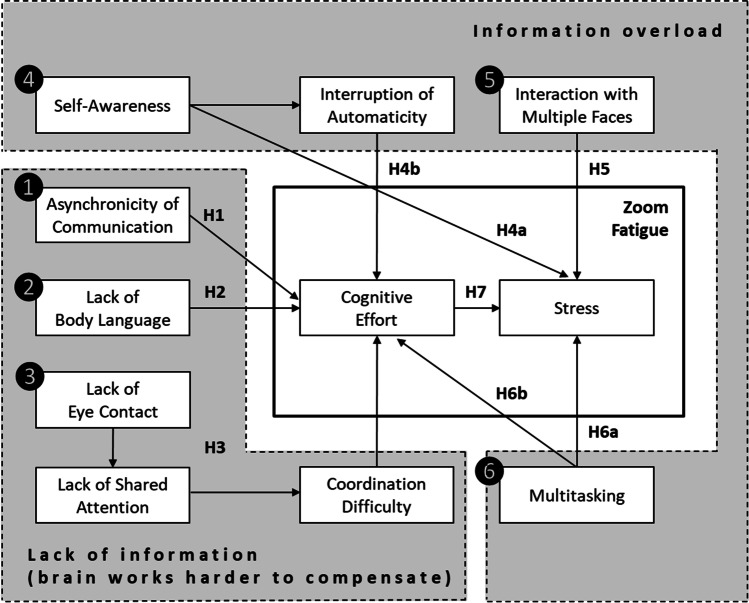
Conceptual framework on the root causes of Zoom fatigue as derived based on media naturalness theory

The following discussion is structured into six subsections (5.1–5.6), each of which deals with a different root cause of Zoom fatigue. In Sect. 5.7, a conceptual framework integrates the six root causes into a theoretical model (Fig. 4) and formulates the resulting hypotheses.

### Asynchronicity of communication

Based on an analysis of empirical research, Federman (2006) argues that “more must be said in a videoconference environment to convey the same meaning, compared to telephone or face-to-face” (p. 440). A number of the existing reports on Zoom fatigue provide an interesting conjecture as to why videoconferencing increases communication ambiguity and cognitive effort. Wiederhold (2020), for example, argues that humans apply a repertoire of “*precisely timed* vocalizations, gestures, and movements to communicate, and they rely on precise responses from others to determine if they are being understood” (p. 437, italics added). Lee (2020) confirms this view and indicates that humans “engage in reciprocal communication, *all in a matter of milliseconds*” (p. 3, italics added).

Brain imaging evidence supports Wiederhold’s (2020) and Lee's (2020) rationale. In a functional magnetic resonance imaging (fMRI) study, Kohrs et al. (2016) compared brain activation patterns in experimental conditions of delays in the range from 200 to 700 ms with immediate feedback. They found that “delays interrupt the course of an interaction and trigger an orienting response that in turn activates brain regions of action control” (p. 1). Moreover, they report that the strength of activation increases with the duration of the delay. In a similar experiment based on functional near-infrared spectroscopy (fNIRS) and skin conductance measurement (Hirshfield et al., 2014), computer response time was manipulated in order to study cognitive effects and arousal. Among other results, it is reported that “subjects simply became frustrated by the manipulation, and their brain activity showed this increase in cognitive load and the need for emotion regulation that is associated with frustration” (Hirshfield et al., 2014, p. 10). Moreover, it was found that the response time manipulation increased arousal, a well well-known manifestation of physiological stress (e.g., Melamed et al., 1999).

Against the background of the presented results, we can theorize as follows: **If a delay is perceived during videoconferencing (even if this perception occurs subconsciously in the range of milliseconds), the human brain works harder and thereby attempts to overcome the issue of asynchronicity, which is accompanied by increased cognitive effort to restore synchrony. Moreover, this effect is likely accompanied by enhanced frustration and stress.**

In general, it can be argued that the human species has a natural tendency to strive for synchrony in communication processes (Stephens et al., 2010), even when that synchrony brings with it energetic costs, such as the cognitive workload caused by maintaining full synchrony in videoconferences. This cost of increased cognitive workload comes along with perception of Zoom fatigue.

### Lack of body language

Lack of emotion recognition via body language is another potential root cause of Zoom fatigue. In this context, researchers conducted an intriguing experiment (Meeren et al., 2005). In the natural world a face is typically encountered as an integrated part of a whole body and not as an isolated object. Hence, in human interactions both face and body convey the emotional state. The study demonstrates that observers judging a facial expression are significantly influenced by emotional body language. To investigate this, the experimenters created face-body compound pictures with either matched or mismatched emotional expressions (using images of faces and bodies, and the emotions of fear and anger). The researchers found that when the face and body convey conflicting emotional information, judgment of facial expressions became more difficult, and hence was biased toward the emotion expressed by the body. Electroencephalography (EEG) data further showed brain alterations as early as 115 ms after stimulus onset, pointing to “the existence of a rapid neural mechanism sensitive to the degree of agreement between simultaneously presented facial and bodily emotional expressions” (p. 16518).

In a similar experiment, Martinez et al. (2016) determined that emotion recognition patterns from the face alone and from the body alone differ as a function of emotion. Specifically, the researchers indicate that angry bodies were more recognizable than angry faces (“when participants saw bodies alone, but not faces alone or faces and bodies, they had a pronounced bias to see these bodies as angry”, p. 948). The researchers’ explanation draws upon evolutionary psychology, the field from which our overarching theory, media naturalness theory, originates (Kock, 2004, 2009a, 2009b). They argue that anger is different from the other emotions because it “represents a direct or imminent threat to the viewer, prompting an increased startle reflex and the ‘fight-or-flight’ response” (p. 948). Because it is advantageous to perceive anger of another individual from a distance via body language rather than by facial expressions, brain structures evolved that highly effectively decode “anger information” from the body (and not solely from facial information). This fact is substantiated by brain imaging studies demonstrating that whole-body expressions of anger correlate with activity in regions which neurologically implement the processing of aversive, emotional, and stressful stimuli (e.g., Sergerie et al., 2008).

**With regard to videoconferencing, one major implication of these research findings is that because videoconference participants normally see only the interaction partners’ faces (but typically not the full body), rapid and accurate emotion perception may be hampered.** This is particularly true for situations in which conflicts could play a role, because anger can become a factor (which would be a significant concern for organizations holding virtual meetings in which, for example, nonperforming projects are discussed or in which high-stake negotiations take place). This increased difficulty of perceiving emotions quickly and accurately increases communication ambiguity and cognitive effort, as well as, eventually, negatively influencing downstream variables such as collaboration satisfaction and effectiveness (Kock, 2004, 2009a, 2009b).

### Lack of eye contact

When people look into each other’s eyes, they experience eye contact. A behavioral study by Richardson et al. (2007) demonstrates that individuals coordinate their attention when synchronously engaging in an interactive dialog. Specifically, it was found that subjects’ eye movements were tightly coupled (i.e., “more likely than chance to be looking at the same thing”, p. 411) in a joint activity task. A brain imaging study by Saito et al. (2010) applied simultaneous fMRI to pairs of subjects who engaged in real-time gaze exchange in a joint attention task. A major result found was that a part of the prefrontal cortex supports the neurological implementation of “sharing intention during eye contact that provides the context for joint attention” (p. 1). Another brain imaging study identifies a hedonic aspect of shared attention (Schilbach et al., 2010). In essence, this study found that self-initiated joint attention in human social interaction results in neural activity in reward-related brain areas. Similar results are reported in other fMRI studies (e.g., Redcay et al., 2010). Altogether, these findings are in line with the “cooperative eye hypothesis”, which states that gaze following in humans has evolved with the purpose of supporting accomplishment of complex tasks that require coordination and cooperation (Tomasello et al., 2007). In other words, **if there is a lack of eye contact, shared attention is more difficult to establish than with eye contact, and this, in turn, leads to coordination difficulty that comes along with increased cognitive effort.**

### Self-awareness

Videoconferencing tools provide people with feedback from their own camera, typically presented as a window on the screen. Thus, when videoconferencing participants look at their screens, they look into a “mirror”, **leading to increased self-awareness**. Bailenson (2021) tellingly describes this effect: “Imagine in the physical workplace, for the entirety of an 8-h workday, an assistant followed you around with a handheld mirror, and for every single task you did and every conversation you had, they made sure you could see your own face in that mirror. This sounds ridiculous, but in essence this is what happens on Zoom calls” (p. 4). Recently, a survey study by Fauville et al. (2021b) found that “mirror anxiety” (captured by a self-report measure and by linguistic analysis of responses to an open-ended question) explains why women experience more Zoom fatigue than men.

The literature predominantly reports negative consequences of self-awareness in communication processes (e.g., Carver & Scheier, 1978; Goffman, 1959; Joinson, 2001). For example, evidence indicates that self-awareness and responsiveness to the evaluation of others are positively correlated (Fenigstein, 1979). A logical idea developing from Fenigstein’s (1979) finding is an understanding that increased self-awareness (triggered by the small window in the interface) leads to a pronounced response to negative evaluations (e.g., criticism that one receives in a videoconference, notably in online education or in an online business meeting). This, in turn, may result in heightened stress reactions and perceptions, as shown by Slavich et al. (2010), whose study found that social evaluative stress is associated with significant increases in markers of inflammatory activity.^6^ Additional neuroimaging evidence in the same study (based on a subsample of the original larger sample) revealed that greater increases in one specific marker were linked to enhanced activity in brain areas related to processing rejection-related distress and negative emotions (Slavich et al., 2010). In addition to this physiological evidence, a self-report study conducted in the workplace context found that subjects’ hypersensitivity to social rejection predicted an increase in stress and burnout (Ronen & Baldwin, 2010). Thus, increased self-awareness enabled by a perception of one’s own videostream is a serious stress factor, contributing to Zoom fatigue.^7^

Another antecedent of Zoom fatigue, complementary to the stress perspective, is an increased self-awareness in human social interaction that **disrupts the automatic processes that are typical for effective communications.** From a cognitive perspective, automatic communication is usually perceived as effortless (e.g., Isbilir et al., 2019). Automaticity means that the interaction partners quickly exchange communicative stimuli (e.g., during videoconferences, this would predominantly be spoken words rather than body language), and they are fully immersed in the conversation. Based on a computer-mediated communication context, Miller et al. (2017) confirm this view. They argue that when one individual is interacting with another and is attending fully to that interaction, “things progress smoothly”. However, if an individual becomes focused on himself or herself, “attention and concern could be shifted away from the interaction itself toward how that interaction will be perceived by others” (p. 5274).

A useful framework for developing a better understanding of automaticity is provided by the X- and C-Systems Theory.^8^ This dual-processing theory (Stanovich & West, 2000) distinguishes automatic and controlled information acquisition, reasoning, and decision-making. The automatic mode is relatively undemanding (from a cognitive capacity perspective), relatively fast, and usually emotionally charged. The controlled mode, in contrast, is more demanding of cognitive capacity, is relatively slow, and is less emotionally charged. Neuroscience research has identified brain regions that correspond more strongly to automatic mental processes (X-System, reflexive), while other areas more strongly correspond to controlled mental processes (C-System, reflective) (for a review, see Lieberman, 2007).

The automatic X-System and its corresponding brain structures are phylogenetically older than the C-System and its structures (Satpute & Lieberman, 2006). This has consequences. First, in many situations automatic processes affect human behavior more than controlled processes do. Second, perception of cognitive effort is higher for controlled than for automatic processes, because both attentional and working memory demands are higher (e.g., Hill & Schneider, 2006). The implication for videoconferencing is that **if a user’s own face is shown on the interface, an automatic communication processes may be disrupted, accompanied by a switch to more controlled mental processes. This, in turn, may result in more pronounced perceptions of cognitive exhaustion and fatigue, due to increased attentional and working memory demands.**

### Unnatural interaction with multiple faces

Many videoconferencing tools use windows to show participants on the screen (see Fig. 2). The extant literature (see examples below) reports that this may lead to a perception that many faces, or their eyes, are staring at an individual. This, in turn, may increase arousal and stress (Senju & Johnson, 2009). Morris (2020), for example, writes: “Images of framed heads of varying sizes are disconcerting, as are the giant faces of speakers. Audiences are particularly sensitive to images of people, especially when they are too big and too close […] activate the sympathetic nervous system associated with the fight-or-flight response—likely in part because they made images look closer and more threatening” (p. 5). Ma (2020) notably confirms this view, arguing that: “When was the last time you held unwavering eye contact with someone for an hour? If we’re in a crowded elevator, we look at the floor. If someone close by is staring, we take a step back. We use different personal space techniques to always maintain an appropriate level of intimacy—which fails to translate online when you’re staring ‘at a huge face inches from your own’” (p. 2).

Bailenson (2021) writes that in a Zoom meeting the size of faces on a screen depends on various factors, such as monitor size, a user’s distance to the monitor, and the number of participants (faces). In a typical use case scenario and assuming a one-on-one meeting and “speaker view” configuration (i.e., own face small window, other person’s face huge window), he tested the face size of the other person and found that the “length from chin to the top of the head of the other person on the screen was about 13 cm” (p. 2). He further argues that this size resembles a situation of “be[ing] about 50 cm away when standing face-to-face [… and] anything below about 60 cm is classified as ‘intimate,’ the type of interpersonal distance patterns reserved for families and loved ones” (p. 2). He also describes a situation with more Zoom participants (comparable to Fig. 2 in the present paper) and compares it to traditional face-to-face meetings. In essence, he indicates that while in the traditional setting many people in the room often do not directly look at the speaker (because they chat with other people or because they direct their gaze somewhere else in the room), in a Zoom meeting the speaker often has the feeling of being stared at and this “causes physiological arousal” (p. 2).

Evidence indicates that humans─without conscious awareness─will typically detect immediately when they are the target of another individual’s gaze (Stein et al., 2011). Being looked at or stared at triggers a bodily response, and this, in turn, is often followed by a behavioral response such as avoidance or withdrawal (Senju & Johnson, 2009). Harrod et al. (2020) review a wealth of empirical studies, both from the domains of human and nonhuman primates, and conclude that direct gaze serves as a signal of threat or dominance, indicating that “physical aggression might soon follow” (p. 1). However, they also summarize papers demonstrating that “eye contact may be used to communicate complex emotional and mental states, and to establish affiliative bonds” (Harrod et al., 2020, p. 1)—an argument previously discussed in Sect. 5.3, where major consequences of lack of eye contact during videoconferences are outlined (i.e., lack of shared attention → coordination difficulty → cognitive effort).

What the literature suggests, if considered in an integrative rather than isolated fashion, is that humans need eye contact in communication processes in order to develop bonding and coordination. Yet, perceptions of unnatural interaction with multiple faces, a frequent phenomenon during videoconferencing (e.g., Bailenson, 2021; Ma, 2020; Morris, 2020), may constitute a source of stress. This stress perception typically triggers bodily stress reactions such as increased activation in brain areas related to arousal, release of stress hormones, blood pressure increase, heart rate increase, and heart rate variability reduction (e.g., Chrousos, 2009; de Kloet et al., 2005). It is important to note that interaction with multiple faces, as well as the resulting feeling of being stared at, do not necessarily imply direct eye contact. The human visual field (i.e., the spatial array of visual sensations available to observation) is approximately 130° vertically and 180°–200° horizontally (Spector, 1990). Thus, developing a perception of being stared at is possible, and even likely, without direct eye contact, only based on interaction with multiple faces.

Study of eye contact in humans has established that the frequency and duration is higher in American and Western European cultures, as compared to East Asian cultures (e.g., Akechi et al., 2013; Blais et al., 2008). Yet, despite slight differences in eye contact tolerance among human cultures, evolution has shaped humans to be able to perceive prolonged eye contact as an implicit signal of threat, associated with “imminent physical aggression” (Harrod et al., 2020, p. 1). This leads to stress (e.g., Seery, 2011), prompting the theory that **unnatural interaction with multiple faces during videoconferencing, including the feeling of being stared at, comes along with increased stress.**

### Multitasking during videoconferences

A frequently observed phenomenon related to videoconferences is that people often engage in other tasks and activities while participating in a videoconference (e.g., Fosslien & Duffy, 2020). Anecdotal evidence effectively describes the phenomenon (Ionos, 2020): “At an in-person meeting, it’s almost impossible to inconspicuously check your emails, make appointments, or send out messages while your co-worker discusses a project. However, if you’re already sitting at your computer for a Zoom call, it’s easy to open up another window or type on your keyboard without anyone noticing” (p. 4). In fact, as outlined in the following, scientific evidence shows that multitasking usually does not increase work productivity; rather, it may even reduce productivity. Moreover, permanent multitasking contributes to stress and fatigue (evidence discussed below).^9^

In a paper entitled “The Myth of Multitasking”, Rosen (2008) presents a gloomy picture about the relationship between multitasking and productivity. She writes that “infomania” (a concept closely related to multitasking, defined as “an effort to miss nothing”) is “a serious threat to workplace productivity” (p. 106). Research with a focus on the cognitive aspects of multitasking also reveals notable results. One study (Ophir et al., 2009) demonstrates that heavy media multitaskers, when compared to light media multitaskers, perform worse on a test of task-switching ability. One explanation for this finding is that heavy media multitaskers are more susceptible to interference from irrelevant stimuli. Using EEG and pupillometry measures of attention, another cognition study found that heavier media multitasking is correlated with a propensity to experience attention lapses and forgetfulness (Madore et al., 2020).

With respect to the effects of multitasking on stress and fatigue, research indicates a positive relationship. One survey study found that higher levels of communication load and multitasking increases perceived stress, which in turn is positively associated with burnout and depression tendencies (Reinecke et al. 2017). Neurophysiological evidence substantiates this survey finding. One examination (Wetherell & Carter, 2014) found that a 15-min period of multitasking (mental arithmetic, auditory monitoring, visual monitoring, and a Stroop task performed on a computer) led to increases in heart rate and systolic and diastolic blood pressure.

The heavy mental workload that results from engagement in activities unrelated to a videoconference session (e.g., checking e-mail) may be further elevated by the requirement to rapidly switch between software features in the tools. Anecdotal evidence indicates that the use of features such as instigating screen sharing, selection of the correct screen to be shared, ending screen sharing, and parallel consideration of comments in the chat can be accompanied by elevated cognitive effort and stress. Imagine, for example, a Zoom meeting in which participants frequently use the chat function to provide comments. The literature on the negative consequences of IT-mediated interruptions reports overwhelming evidence that such interruptions typically cause stress, both self-reported (Tams et al., 2015, 2018) and physiological (Galluch et al., 2015; Riedl et al., 2012; Riedl et al., 2013).

Thus, **the frequently observed behavior of videoconference participants engaging in multiple unrelated activities while in a video session, along with switching between the various software features in the tools and the processing of instant messages, constitutes a root cause of the fatigue and stress felt as a consequence of videoconferencing.**

### Conceptual framework and hypotheses

Figure 4 graphically summarizes the root causes of Zoom fatigue as described in the preceding sections.

*First*, a slight delay during videoconferencing makes the brain work harder to overcome the issue of asynchronicity. The type of delay addressed here includes subconscious delay perceptions in the range of milliseconds. Perception of delays, even in the millisecond range, has a “price”, namely the increased cognitive effort to restore synchrony. *Second*, because videoconference participants normally see only the interaction partners’ faces (but typically not the full body), the brain has to work harder in such situations to overcome this deficit in body language information, causing increased cognitive effort. *Third*, videoconferencing involves lack of eye contact. We theorized that the link to cognitive effort is likely not a direct one. Rather, if there is a lack of eye contact, shared attention is difficult to establish, and this, in turn, leads to coordination difficulty that comes along with increased cognitive effort.^10^ As a complement to Fig. 3, where we conceptualize our theorizing based on media naturalness theory, root causes (1), (2), and (3) are summarized in the “lack of information” area in Fig. 4.

*Fourth*, a well-known feature in videoconferencing interfaces is a window in which a user can see his or her own face. Self-awareness may lead to stress. Moreover, increased self-awareness may disrupt automatic communication processes (“automatic” here means that the communication partners quickly exchange communicative stimuli such as spoken words, and they are fully immersed in the conversation). However, if automaticity is interrupted due to self-awareness, attention is shifted away from the interaction itself toward the way in which an individual is perceived by other videoconference participants. This shift is accompanied by perceptions of increased cognitive effort, due to self-reflections and mentalizing activities. In this context, mentalizing mainly refers to thoughts such as “What do other people think about me?” or “How am I perceived by others?”. *Fifth*, during videoconferences we often see the faces of many other participants. Therefore, participants often develop a feeling that they are the target of other individuals’ gazes. Being looked at or stared at triggers bodily responses related to threat and stress. Despite the fact that culture, social norms, and situational factors (e.g., familiarity with the communication partners) might attenuate the negative effects that being stared at imposes on stress, evolution has “programmed” humans to perceive an implicit, and hence hardly avoidable, signal of threat that accompanies a stress response. Thus, interacting with multiple faces during videoconferences is a source of stress. *Sixth*, a frequently observed phenomenon is that videoconference participants engage in multiple cognitive activities that are unrelated to the actual video session (e.g., checking e-mails, posting and sharing on social media, reading online news), along with switching between the various software features in the tools (e.g., starting and ending of screensharing) and the processing of instant messages. It follows that people deal with many different things during a video session. Therefore, people often multitask during videoconferences, and this has consequences. Evidence indicates that multitasking may have severe negative effects on stress and fatigue, as well as on related symptoms (e.g., emotional exhaustion, burnout, and depressive tendencies). These effects are likely mediated by increased cognitive effort. As a complement to our rationale in Fig. 3, the root causes (4), (5), and (6) are summarized in the “information overload” area in Fig. 4.

Moreover, as indicated in the Fig. 4 solid rectangle, cognitive effort and stress have been shown to be distinct constructs, and typically the former precedes the latter (e.g., Hjortskov et al., 2004; Hockey, 1997; Irie et al., 2001; Mandrick et al., 2016; Parent et al., 2019; Peters et al., 1998). This conceptualization also implies that Zoom fatigue, based on the theorizing in the present paper, does have two distinct, yet related, dimensions: cognitive effort and stress. This view is not only consistent with the conceptualization and definition of the phenomenon that was developed in Sect. 3 based on a systematic review of the literature, but it is also in line with a recent survey instrument to measure “Zoom exhaustion and fatigue” because this instrument includes stress-related items such as “I fell emotionally drained” or “I feel irritable” (Fauville et al., 2021a, p. 9). Table 3 summarizes the hypotheses that result from the conceptual framework.

**Table 3:** Summary of hypotheses based on the conceptual framework

Hypotheses (based on conceptual framework, Fig. 4)	Major sources
H1: Transmission delay of videoconferencing tools increases asynchronicity of communication which, in turn, increases cognitive effort	Lee (2020), Kohrs et al. (2016), Richardson et al. (2007), Rump and Brandt (2020a), Saito et al. (2010), Schilbach et al. (2010)
H2: Lack of body language perception during videoconferencing increases cognitive effort	Bailenson (2021), Fauville et al. (2021b), Kock (2005), Kock (2009a), Kock (2009b)
H3: Lack of eye contact during videoconferencing increases cognitive effort. This relationship is mediated by lack of shared attention and resulting coordination difficulty	Redcay et al. (2010), Richardson et al. (2007), Saito et al. (2010), Schilbach et al. (2010), Tomasello et al. (2007)
H4: Display of a user’s own face during videoconferencing increases self-awareness, which, in turn, increases (H4a) stress, and (H4b) disrupts automaticity in information processing, causing increased cognitive effort	Bailenson (2021), Carver and Scheier (1978), Fenigstein (1979), Joinson (2001), Lieberman (2007), Miller et al. (2017), de Guinea and Webster (2013)
H5: Interaction with multiple faces during videoconferencing leads to stress	Akechi et al. (2013), Bailenson (2021), Blais et al. (2008), Fauville et al. (2021b), Harrod et al. (2020), Ma (2020), Morris (2020), Senju and Johnson (2009), Seery (2011)
H6: Multitasking during videoconferencing increases (H6a) stress directly, and (H6b) indirectly via cognitive effort	Fosslien and Duffy (2020), Ionos (2020), Madore et al. (2020), Ophir et al. (2009), Reinecke et al. (2017), Rosen (2008), Wetherell and Carter (2014)
H7: Cognitive effort influences stress	Hjortskov et al. (2004), Hockey (1997), Irie et al. (2001), Mandrick et al. (2016), Parent et al. (2019), Peters et al. (1998)

## Discussion

### Implications for research

Section 5, based on media naturalness theory as overarching model, integrates both behavioral and neuroscience literature from various scientific disciplines in order to develop a conceptual framework on the root causes of Zoom fatigue. However, the fact that this framework and its inherent hypotheses were carefully developed based on a solid theoretical foundation cannot substitute for original empirical research. Accordingly, future studies should apply different videoconferencing contexts (e.g., business, online education) in order to empirically examine the framework and hypotheses.

How should the conceptual framework be tested in future studies? Different research strategies are possible. First, if an experimental approach is applied, one does not need to test the complete model in one single experiment. The number of resulting conditions would require a very large sample size.^11^ Therefore, it is more realistic that future experiments test specific parts of the model. If an experimental approach is applied, we recommend the complementary use of self-report and neurophysiological measurement where possible, because both kinds of data often tap into different aspects of a construct, which is particularly true for the two dimensions of Zoom fatigue, namely cognitive effort (e.g., Rubio et al., 2004) and stress (e.g., Tams et al., 2014).^12^ In addition to experiments, survey studies should be conducted to test the framework and its underlying hypotheses. Finally, even qualitative methods (e.g., based on interviews) could be appropriate to examine the theoretical frame. In doing so, the constructs in the conceptual framework would serve as categories for coding of the collected data.

Theoretical frameworks should be parsimonious. However, they should also explain a large variance in the outcome variable, here Zoom fatigue. Independent of the specific findings of future studies, it is clear that some variance will not be explained by the constructs in the theoretical model (Fig. 4). Thus, it is definite that Zoom fatigue is affected by additional variables, and future research should therefore revise and advance the framework.

The implicit assumption of the present paper is that videoconferencing stress is a dark side phenomenon. It follows that the present conceptualization of the phenomenon draws upon a distress (i.e., stress that creates a threat or hindrance) rather than eustress (i.e., stress that creates a challenge or an opportunity) perspective. However, recently Tarfardar et al. (2019) made a call for more studies on eustress because “not all stressors are detrimental to the individual” (p. 12) and because “[t]echnostress is experienced differentially by the individual, depending on whether IS characteristics are appraised as challenge or threat stressors” (p. 25). But what is the implication of this “eustress” perspective for videoconference stress research? We should revisit the constructs in the conceptual framework and the corresponding hypotheses and ask whether it is possible that the mechanisms at play are eventually not valid for all people or situations. Consider the following example.

In our framework we hypothesize that the frequently observed behavior of videoconference participants engaging in multiple unrelated activities while in a video session (multitasking) constitutes a root cause of Zoom fatigue. Interestingly, despite the fact that Zoom fatigue was unknown at the time Tarafdar et al. (2019) published their research agenda paper, it seems that they had a premonition of the phenomenon because they wrote about “productive multi‐tasking during meetings when the individual is not directly contributing or speaking, by working simultaneously on other IS‐mediated tasks” (p. 18). In fact, despite outstanding empirical evidence, it is possible that multitasking during videoconferences might *reduce* people’s overall work stress. This holds true at least for the people whose workload is so high that they have to accomplish additional tasks during video sessions in order to get their work done (assuming that they are not the main speaker). Therefore, while the presented framework constitutes one way to conceptualize videoconferencing (i.e., distress view), alternative ways exist (such as the eustress perspective). Examining these alternative perspectives is an important future research endeavor in order to generate a more complete picture of the stress potential of videoconferencing.

Even if one stays within the “distress” and “hindrance stressors” perspective, complementary conceptualizations to the one discussed in the present paper exist in order to determine the stress potential of videoconferencing. Consider a recent article by Benlian (2020), who outlines six “hindrance stressor facets” (p. 1264): (1) obstacle (system failure, breakdown), (2) constraint (missing features, usefulness issue), (3) source of ambiguity (insufficient use skills), (4) habit breaker (work routines must be changed), (5) invader (accessible anytime/anyplace), and (6) interrupter (task continuity is distracted). This taxonomy can be applied directly to the study of videoconferencing stress. Future research does not necessarily need to address all six facets in one single study. Rather, specific foci are possible. As an example, because videoconferencing tools are frequently used in the home office, the important question arises whether the technology is perceived as invader. Recent evidence (DeFilippis et al., 2020; Gimpel et al., 2020) supports the idea that videoconferencing is likely perceived as invader by many people worldwide. Benlian’s (2020) landmark publication on technology-driven spillover effects from work to home could serve as a starting point for future investigations.

### Implications for practice

Based on the Zoom fatigue literature summarized in the Appendix, 17 coping strategies could be identified. Table 4 summarizes the 17 strategies. The author of the present paper has grouped the 17 strategies into three categories: organizational countermeasures (etiquette), personal behavioral rules to avoid stress, and use of software features designed to imitate F2F interaction.

**Table 4:** Summary of coping strategies

Coping strategies for Zoom fatigue	Sample sources	Sample research questions (RQ)
*Organizational countermeasures*		
Having audio-only conferences/turning off the camera/reducing onscreen stimuli to avoid overstimulation / using telephone calls as alternative	Brown Epstein (2020), Fosslien and Duffy (2020), Ionos (2020), Ma (2020), Rump and Brandt (2020a), Sklar (2020), Wiederhold (2020)	RQ 1: Do audio-only meetings, or telephone calls, cause less cognitive effort than videoconferencing?
Booking virtual meetings at least 24 h in advance so that participants have time to prepare/timely agenda setting	Ma (2020)	RQ 2: Does avoidance of short-term booking of videoconferences reduce stress?
Scheduling breaks between virtual meetings	Brown Epstein (2020), Fosslien and Duffy (2020), Ma (2020), Rump and Brandt (2020a), Wiederhold (2020)	RQ 3: How do breaks contribute to recovery from the negative effects of videoconferences, and which break designs are most effective?
Holding fewer virtual meetings/limiting use of videoconferencing tools	Ionos (2020), Wiederhold (2020)	RQ 4: Does limiting the number of videoconferences per day, or per week, reduce stress?
Limiting the meeting time	Ionos (2020), Rump and Brandt (2020a)	RQ 5: Does limiting the meeting time of a videoconference (e.g., 30, 45, or 60 min) reduce mental workload and stress?
Advising that participants use mute when not speaking (to avoid interruptions)	Wiederhold (2020)	RQ 6: Does muting reduce cognitive effort and stress during videoconferencing?
Varying activities / involving the audience with a poll or questions (e.g., in online lectures)	Brown Epstein (2020), Toney et al. (2021)	RQ 7: Does regular change of stimuli during videoconferencing reduce cognitive effort and stress?
*Personal Behavioral Rules*		
Avoid multitasking	Brown Epstein (2020), Fosslien and Duffy (2020), Wiederhold (2020)	RQ 8: What forms of multitasking do people apply during videoconferencing, do other participants perceive their partners’ multitasking, and what are the stress and group performance effects?
Become comfortable with the software	Brown Epstein (2020)	RQ 9: Do videoconferencing tool literacy and computer self-efficacy reduce stress?
Have a good infrastructure (e.g., strong Internet connection)	Brown Epstein (2020), Fouda (2020)	RQ 10: What Internet connection (bandwidth) do people use for videoconferencing, and is this correlated with cognitive effort and stress?
Be sure that faces are lighted from the front (making it easier to see microexpressions)	Brown Epstein (2020), Wiederhold (2020)	RQ 11: Does perception of the interaction partners’ facial features alter perceived stress?
Log in early	Brown Epstein (2020)	RQ 12: Do last-minute logins affect stress?
Stand up and stretch during the session	Brown Epstein (2020)	RQ 13: Do changes of body positions during videoconferences affect stress?
*Software Features to Imitate Face-to-Face Interaction*		
Use software tools that create spatial faithfulness (the extent to which the tool preserves spatial relationships)	Nguyen and Canny (2007)	RQ 14: Which software tools offer features that create spatial faithfulness, do people know about them, and do these features affect cognitive effort?
Use software tools that offer a “together mode” to create the perception that all participants are in the same room and share the same context	Brown Epstein (2020), Rump and Brandt (2020a)	RQ 15: Does use of the “together mode” (e.g., Microsoft Teams) affect perception of social presence, and does this perception affect stress?
Use software tools with an attention-correction function so that gaze is automatically corrected toward the camera, to support perception of eye contact/use tools that correct participants’ gaze direction	Rump and Brandt (2020a)	RQ 16: Does perception of eye contact during videoconferencing increase stress perceptions?
Use smartphones (not desktop versions of videoconferencing tools), because the screen and camera are close together, supporting perception of eye contact	Bekkering and Shim (2006)	RQ 17: What are the cognitive effort and stress implications of using different devices (e.g., smartphone vs. desktop PC) to participate in a videoconference?

To the best of the author’s knowledge, no scientific study to date has empirically evaluated the efficacy of these strategies. Future studies should close this gap and therefore important research questions are formulated in Table 4. However, despite the pending empirical test of these research questions (RQ), practitioners should consider the recommendations in Table 4 (all of which have been derived based on conceptual argumentation rather than empirical research). For example, the first recommendation is to at least sometimes turn off the camera during videoconferencing, thereby reducing onscreen stimuli to avoid overstimulation. The speech imperative proposition, a major element of media naturalness theory (Kock, 2009a), is an important foundation for this advice. This proposition states that “suppressing the ability to convey and listen to speech would substantially affect the naturalness of a medium, more than suppressing the ability to use facial expressions and body language, which should in turn be observed in variables directly or indirectly associated with cognitive effort” (Kock, 2004, p. 335). Therefore, despite pending empirical evidence on RQ1, it is likely that following the advice constitutes an effective countermeasure against fatigue and stress, at least to some extent.

As another example, to the best of the author’s knowledge, no empirical study to date has explored the efficacy of breaks during videoconferencing (see RQ 3). However, research in human–computer interaction has already revealed useful insights that should be considered by practitioners. In essence, based on heart rate variability, electrodermal activity, and blood pressure evidence it has been demonstrated that a 10 min break during longer interaction with digital technologies may effectively reduce user stress (Boucsein & Thum, 1997; Hjortskov et al., 2004). This finding has two implications for videoconferencing. First, longer video sessions such as online lectures should have breaks of approximately 10 min every hour. Second, short breaks should be made between consecutive virtual meetings. In the business context, it can be frequently observed that the end of one virtual meeting comes along with the immediate start of the next meeting. Recent research refers to this phenomenon of missing or little time between two consecutive virtual meetings as burstiness, and it has already been shown that burstiness and videoconference fatigue are positively correlated (Fauville et al., 2021b).

From a practitioner perspective, it is also of utmost importance to consider the *health implications* of Zoom fatigue. Recent survey evidence (Rump & Brandt, 2020a) found that Zoom fatigue manifests itself in various medical symptoms. Specifically, 30% of respondents who already experienced Zoom fatigue reported headache, 28% back pain, 23% visual disorders, and 14% insomnia. Because these symptoms typically come along with alterations in the body (e.g., increased levels of cortisol, Melamed et al., 1999), it is critical that future studies deal with the medical consequences of the fatigue and stress that result from videoconferencing. Despite the current paucity of corresponding research due to the novelty of the phenomenon, it is clear that practitioners should actively prevent, or at least mitigate, the possible negative stress-related consequences. The coping strategies in Table 4 are a starting point. Also, the debate should be continued regarding whether Zoom fatigue should eventually be considered as a stress-related disorder in future international diagnostic classifications (e.g., Anderson & Looi, 2020).

Finally, practitioners should also consider the *cognitive implications* with respect to reasoning processes and decision making. In the present paper, we discussed evidence indicating that videoconferencing may lead to higher levels of cognitive effort, if compared to F2F interaction. According to the seminal heuristic systematic model (HSM) developed by Chaiken (1980; Chaiken & Eagly, 1983; Chaiken et al., 1989), heuristic processing takes precedence over systematic processing when available cognitive resources are limited. What follows is that one major consequence of videoconferencing might be that reasoning and decision change from being systematic to being heuristic. Based on a field study, Ferran and Watts (2008) investigated this hypothesis and confirmed it. Specifically, they found that individuals participating in a seminar via videoconference were more influenced by the likeability of the speaker than by the quality of the presented arguments, whereas the opposite pattern was found for participants attending an in-person seminar. Importantly, differences in cognitive load (measured via a 3-item survey) explain these effects. This conclusion has far-reaching practical implications, as it demonstrates that the mode of communication significantly affects human information processing patterns and resulting behavioral consequences. In the business context, the possible consequences of virtual board meetings, video sessions of virtual project teams, or negotiations with suppliers and customers are notable. Likeability of the speaker as a factor that determines decisions, rather than quality of argument as the determining factor, would likely have adverse consequences for prosperous long-term development of economy. The same holds true for science and education. Imagining virtual scientific conferences or distance learning where the quality of argument cannot be processed properly due to limitations of cognitive resources that result from videoconference fatigue readily reveals the potential for a fatal scenario.

## Concluding statement

Several decades ago the American historian Melvin Kranzberg formulated a set of laws for technology, the first of which states: *“Technology is neither good nor bad, nor is it neutral.”* (Kranzberg, 1986). In fact, it is never easy to predict the unintended consequences and effects of adopting a technology. However, it is the duty of science to develop a sound basis, including theory and evidence, to inform the design of effective interventions against the possible negative effects of technology use. With regard to the current concern for the fatigue and stress resulting from videoconference use, it will be rewarding to see what insights future research will reveal. The present article contributes to this upcoming research field with a carefully derived definition of the phenomenon and a conceptual framework on the root causes of Zoom fatigue which is based on media naturalness theory.

## Appendix: Results of literature review on Zoom fatigue

This table summarizes the papers which we identified based on a systematic literature review via Web of Science, Scopus, Harzing’s software (version 7.31 Windows GUI edition) based on data source Google scholar, AIS eLibrary, ACM Digital Library, and IEEE Xplore (last query: May 2, 2021).

**Table:**

			Step 1		Step 2	Step 3							
*No*	Reference	Publication Date	WOS	SCO	HAR	AIS	ACM	IEEE	Publication Type	Peer-reviewed?	Method	Author Background	Definition
1	Degges-White (2020)	April 4, 2020			x				Online report	No	–	Academic	–
2	Petriglieri (2020)	April 4, 2020	x	x	x				Dialogues	No	–	Academic	–
3	Sacasas (2020)	April 21, 2020			x				Online report	No	–	Writer	–
4	Jiang (2020)	April 22, 2020			x				Online report	No	–	Journalist	–
5	Miller (2020)	April 23, 2020			x				Online report	No	–	Reporter	x
6	Sklar (2020)	April 24, 2020			x				Online report	No	–	Journalist	–
7	Fosslien and Duffy (2020)	April 29, 2020			x				Online report	No	–	Consultant	–
8	Robert (2020)	April 30, 2020			x				Online report	No	–	Reporter	–
9	Schroeder (2020)	May 6, 2020			x				Online report	No	–	Academic	x
10	Callahan (2020)	May 11, 2020			x				Online report	No	–	Reporter	–
11	Hines and Sun (2020)	May 11, 2020			x				Online report	No	–	Academic	x
12	Sander & Bauman (2020)	May 19, 2020			x				Online report	No	–	Academic	–
13	Dixon-Saxon (2020)	May 26, 2020							Presentation slides	No	–	Academic	x
14	Daigle (2020)	May 27, 2020			x				Online report	No	–	Reporter	–
15	Lee (2020)	June 27, 2020			x				Online report	No	–	Academic	x
16	Williamson (2020)	July 2020		x					Opinion	No	–	Consultant	–
17	Maheu and Wright (2020)	July 6, 2020			x				Online report	No	–	Academic	–
18	Basu (2020)	July 9, 2020			x				Online report	No	–	Reporter	–
19	Wiederhold (2020)	July 10, 2020	x	x	x				Editorial (by EIC)	No	–	Academic	x
20	Chrisman (2020)	July 12, 2020		x					Debate	No	–	Academic	–
21	Bothra (2020)	July 20, 2020			x				Online report	No	–	Therapist	–
22	Pesce (2020)	July 28, 2020		x				x	Crosstalk	No	–	Academic	–
23	Tufvesson (2020)	August 10, 2020			x				Online report	No	–	Journalist	–
24	Rump and Brandt (2020a)	September 2020							Research report	No	Survey	Academic	x
25	Cranford (2020)	September 2, 2020		x	x				Editorial (by EIC)	No	–	Academic	–
26	Panke (2020)	October 2, 2020			x				Online report	No	–	Reporter	–
27	Anderson and Looi (2020)	October 5, 2020							Correspondence to editor	No	–	Academic	x
28	Ebner and Greenberg (2020)	October 6, 2020	x	x					Research article	Yes	Conceptual	Academic	x
29	Fernandes (2020)	October 14, 2020		x					Online report	No	–	Analyst	–
30	Collins (2020)	October 21, 2020	x	x					Research article	Yes	Interviews, Observation	Academic	–
31	Peper & Yang (2020)	November 24, 2020			x				Online report	No	–	Academic	–
32	Hall (2020)	November 25, 2020		x					Correspondence to editor	No	–	Academic	–
33	Nadler (2020)	December 2020		x	x				Research article	Yes	Conceptual	Academic	x
34	Schroeder (2021)	January 20, 2021			x				Online report	No	–	Academic	–
35	Toney et al. (2021)	February 2, 2021		x	x	x			Research article	Yes	Case study	Academic	–
36	Chawla (2021)	February 4, 2021	x	x	x				Correspondence to editor	No	Survey	Journalist	–
37	Abdelrahman (2021)	February 5, 2021	x	x					Research article	Yes	Conceptual	Academic	x
38	Bailenson (2021)	February 23, 2021			x				Invited paper	No	Conceptual	Academic	–
39	Fauville et al. (2021a)	February 23, 2021			x				SSRN paper	No	Survey	Academic	x
40	Kageyama (2021)	After February 23, 2021			x				Online report	No	–	Academic	–
41	Palti and Rosenberg-Kima (2021)	March 2021					x		Proceedings	Yes	Field study	Academic	–
42	Wong (2021)	March 18, 2021			x				Online report	No	–	Academic	–
43	Peper et al. (2021)	March 29, 2021			x				Research article	Yes	Survey	Academic	–
44	Williams (2021)	April 9, 2021			x				Correspondence to editor	No	–	Academic	–
45	Fauville et al. (2021b)	April 14, 2021			x				SSRN paper	No	Survey	Academic	(x)

Order of articles by publication date. In five cases (no. 16, 24, 33, 40, 41) identification of the exact publication day was not possible. For no. 16, 24, 33, and 41 only the publication month was available, for no. 40 no information was available; yet, based on analysis of references it was possible to determine the earliest possible publication date. Method column: “–“ indicates that no method was identifiable. Note that the definition in Fauville et al. (2021b) is almost identical to the definition in Fauville et al. (2021a); therefore, despite that 13 papers listed in this table provide a formal definition for the term “Zoom fatigue”, only 12 unique definitions could be identified (listed in Table 1 in the main paper). Database acronyms: WOS = Web of Science, SCO = Scopus, HAR = Harzing’s software (based on Google Scholar), AIS = AIS eLibrary, ACM = ACM Digital Library, IEEE = IEEE Xplore. Indication Step_1_, Step_2_, and Step_3_ in the top row of the table refers to the steps in the literature search process as described in the main paper

## Footnotes

1 Zoom Video Communications, Inc., is the market leader for web-based videoconferencing, and already had 300 million daily meeting participants at the end of April 2020—that is, shortly after the announcement of the first wave of lockdowns (Warren 2020). In March 2021, *zoom.us* had 2.72 billion website visits (Statista 2021a, b). Other tools are, for example, Microsoft Teams and Cisco Webex.

2 Considering the characteristics of different review types as outlined in Paré et al. (2015) and Schryen et al. (2017, 2020), the present literature analysis constitutes a descriptive review.

3 Specification was title; databases: Web of Science, Scopus, and Google Scholar based on Harzing’s “Publish or Perish” software (version 7.31 Windows GUI edition). Note that based on these keywords Web of Science yielded 9 results, Scopus 13 results, and Harzing’s 0 results. However, the identified papers predominantly deal with utilization of telehealth (i.e., videoconferencing) to treat stress disorder patients. As an example, please see Germain et al. (2009). What follows is that these papers do *not* deal with the stress and fatigue potential of videoconferencing as mode of communication.

4 Note that the definition in one further paper, namely in Fauville et al. (2021b), is almost identical to the definition in Fauville et al. (2021a) and hence was not added to the list of definitions.

5 Note that the definitions #7 and #12 are based on Wolf (2020) who focuses on videoconferencing and stress.

6 The markers are: sTNFαRII: soluble receptor for tumor necrosis factor-α. IL-6: interleukin-6.

7 Note that Fenigstein’s (1979) theoretical account also predicts that increased self-awareness may result in increased positive perceptions (e.g., feelings of pleasure) if an evaluation of others is positive. However, at least in the business context, it is difficult to imagine that praise by others is a frequent phenomenon in videoconferencing.

8 In this context, also see Kahneman’s (2011) work “Thinking, Fast and Slow”, which describes a dichotomy between two modes of thought: System 1 is instinctive, emotional, and hence fast, while System 2 is more deliberative, more logical, and hence slower.

9 Multitasking is defined as “attending to or acting on multiple stimuli, activities, or interactions simultaneously” (O’Leary et al., 2011).

10 The author would like to thank an anonymous reviewer for suggesting this mediation effect via coordination difficulty.

11 Imagine a between-subjects design and that each of the six independent variables (root causes) would only have two values (e.g., asynchronicity of communication: 100 ms vs. 500 ms latency time). This factorial design would result in 2^6^ = 64 conditions. Assuming at least 15 subjects in each condition, the necessary sample size would be 960 subjects.

12 The neuroscience tools applied in future studies should range from measurement of brain activity (e.g., fMRI, fNIRS, EEG), evaluation of autonomic nervous system activity (e.g., heart rate and heart rate variability, skin conductance, pupil dilation), to hormone measures (e.g., adrenaline, noradrenaline, cortisol), including additional substances such as alpha amylase.
